# Pigs lacking the SRCR5 domain of CD163 protein demonstrate heritable resistance to the PRRS virus and no changes in animal performance from birth to maturity

**DOI:** 10.3389/fgeed.2024.1322012

**Published:** 2024-03-13

**Authors:** Clint Nesbitt, Lucina Galina Pantoja, Benjamin Beaton, Ching-Yi Chen, Matt Culbertson, Perry Harms, Justin Holl, Andrzej Sosnicki, Srinu Reddy, Marisa Rotolo, Elena Rice

**Affiliations:** ^1^ Genus plc Research and Development, DeForest, WI, United States; ^2^ Genus plc PIC, Hendersonville, TN, United States

**Keywords:** pigs, porcine, PRRS, gene editing, CRISPR, disease resistance

## Abstract

Porcine reproductive and respiratory syndrome (PRRS) is one of the world’s most persistent viral pig diseases, with a significant economic impact on the pig industry. PRRS affects pigs of all ages, causing late-term abortions and stillbirths in sows, respiratory disease in piglets, and increased susceptibility to secondary bacterial infection with a high mortality rate. PRRS disease is caused by a positive single-stranded RNA PRRS virus (PRRSV), which has a narrow host-cell tropism limited to monocyte–macrophage lineage cells. Several studies demonstrated that the removal of CD163 protein or, as a minimum, its scavenger receptor cysteine-rich domain 5 (SRCR5) precludes the viral genome release, conferring resistance to PRRSV in live animals. Today, very limited information exists about the impact of such edits on animal performance from birth to maturity in pigs. Using CRISPR–Cas9 with dual-guide RNAs and non-homologous end joining (NHEJ), first-generation (E0) pigs were produced with a deletion of exon 7 in the *CD163* gene. The selected pigs were bred to produce the next three generations of pigs to establish multiple lines of pigs homozygous for the edited allele, thereby confirming that the *CD163* gene with removed exon 7 was stable during multiple breeding cycles. The pigs were evaluated relative to non-edited pigs from birth to maturity, including any potential changes in meat composition and resistance to PRRSV. This study demonstrates that removing the SRCR5 domain from the CD163 protein confers resistance to PRRSV and, relative to unedited pigs, resulted in no detected differences in meat composition and no changes in the growth rate, health, and ability to farrow. Together, these results support the targeted use of gene editing in livestock animals to address significant diseases without adversely impacting the health and well-being of the animals or the food products derived from them.

## 1 Introduction

Porcine reproductive and respiratory syndrome (PRRS) emerged in the 1980s in the US and has spread rapidly worldwide. During the last 30 years, despite many vaccines developed against the causative agent PRRS virus (PRRSV), PRRS continues to have a significant economic impact on the pig industry (Holtkamp et al., 2013). Clinical signs vary among herds due to the overall health status, differences in isolate virulence, and management practices. The most common initial symptoms of the disease include the loss of appetite, lethargy, and depression. In acute cases, these signs progress to premature farrowing and an increased number of stillborn pigs with a significant increase in preweaning mortality. Growing pigs infected with PRRSV may have respiratory symptoms and secondary infections, show slow growth, and reduced weight (Christianson and Joo, 1994). Today, more than 60% of sow farms in the US can be PRRS-positive^1^, leading to a significant economic impact due to added health and biosecurity measures of up to $9.54 per pig (Holtkamp et al., 2013). More recent data suggest that depending on the type of the farm (boar stud, finishing, nursery, and sow) and the virulence of the isolate (low, mid, and high), the economic losses may vary between 0.5M USD and 16M USD per farm (Valdes-Donoso and Jarvis, 2022). Because PRRSV suppresses the immune system of pigs, worsening the clinical impacts of any bacterial infections, increased antibiotic use is needed to protect the health of affected pigs.

Despite a long history of PRRS disease, existent vaccines against PRRSV are only partially effective against infections and may reduce clinical symptoms and the intensity of the outbreaks (Nan et al., 2017). Attempts to increase resistance against the PRRS virus through selection and breeding have not been successful, and a native allele conferring tolerance to the virus has not been identified in current populations (Johnsson et al., 2018). As a result of intensive basic research focused on the virus entry into host cells and its primary target cells, pulmonary alveolar macrophages (PAMs), many cellular factors involved in virus binding, internalization, and genome release have been identified (Ma et al., 2022). The CD163 protein, a scavenger receptor cysteine-rich (SRCR) family for hemoglobin clearance, was demonstrated as the most specific and indispensable receptor for PRRSV entry and infection (Calvert et al., 2007). The CD163 protein is a membrane protein comprising nine SRCR domains, where the SRCR5 domain was identified as the critical domain for PRRSV infection with no involvement from other CD163 SRCR domains (Van Gorp et al., 2010a). With the development of gene editing technology, several edited versions of the *CD163* gene in pigs have been developed and shown to confer complete resistance to infection caused by PRRS viruses (Whitworth et al., 2016; Burkard et al., 2018; Yang et al., 2018; Guo et al., 2019). The *CD163* variants tested in pigs included a complete knockout of *CD163* (Whitworth et al., 2016; Yang et al., 2018), the removal of a 41aa fragment of the SRCR5 domain (Guo et al., 2019), and the removal of the whole SRCR5 domain, leaving the rest of the protein intact (Burkard et al., 2018). Recently, Stoian et al. (2022) demonstrated specific amino acid sequences within the SRCR5 domain that is critical for uncoating both type 1 and type 2 PRRS virus isolates. Considering that CD163 has a variety of essential biological functions (Van Gorp et al., 2010b), the removal of only the SRCR5 domain of CD163, which seems to not have any other known function besides the interaction with the PRRS virus, while maintaining a stable expression of the residual domains of CD163, can minimize any potential negative impact on pigs’ growth, development, and robustness. Recently, Burger et al. (2024) described the introduction of a single modified *CD163* allele with an SRCR5 domain removed in four diverse, genetically elite porcine lines without any off-target edits. Here, we report further comprehensive testing of the conventionally bred pigs with edited *CD163* alleles for the first time. Our data demonstrate that removing the SRCR5 domain confers complete resistance to the current PRRS virus isolates. The results of phenotypical evaluation from birth to finishing and reproductive phases show no differences between edited and control pigs. No differences in meat quality and composition were observed as well. In summary, pigs with modified CD163 protein are not different from the control pigs, except for the resistance to the infection caused by the PRRS virus^2^.

## 2 Materials and methods

### 2.1 Generation and breeding of pigs with the CD163ΔE7 edit

**FIGURE 1:**
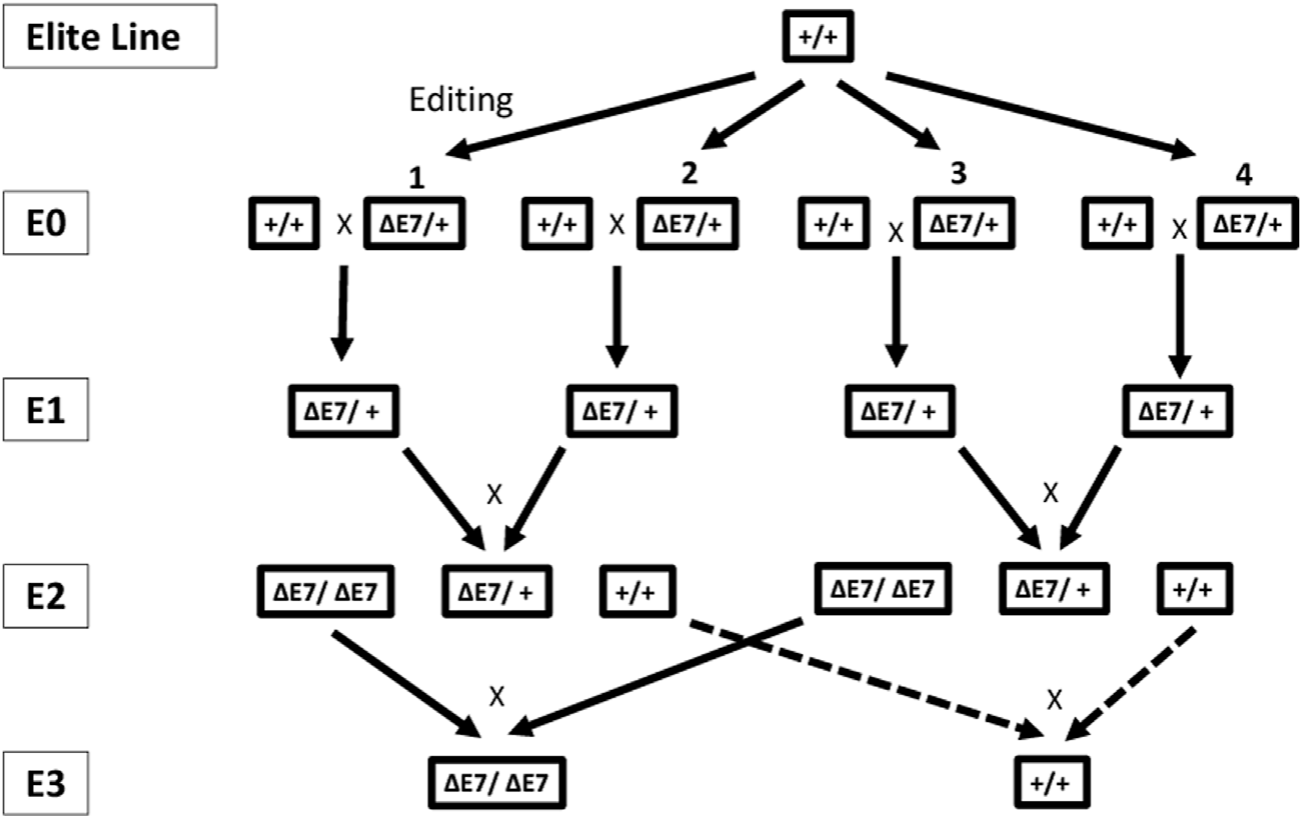
Breeding approach to develop the multiple generations of edited pigs for evaluation of disease resistance, phenotypical characteristics, and meat composition and quality. Multiple edited E0 animals (*CD163* ^ΔE7/+^) from the same elite breeding line were crossed with unedited, wild-type animals (*CD163* ^+/+^) to produce the heterozygous (*CD163* ^ΔE7/+^) E1 generation. The segregating E2 population was created by interbreeding among E1 animals. Homozygous-edited (*CD163* ^ΔE7/ΔE7^) and null (*CD163* ^+/+^) animals in the E3 generation derived from crosses between homozygous-edited and null E2 animals, respectively.

The first-generation (E0) pigs were produced using a zygote injection with a SpCas9/gRNAs complex, as described in Burger et al. (2024). The E0-edited pigs were screened to identify pigs containing the edited *CD163* allele where exon 7 encoding the SRCR5 domain was removed, and the resulting sequence was identical in all selected pigs, as described in Burger et al. (2024). Boars with the edited *CD163* allele (*CD163* ^ΔE7/+^) were mated with corresponding wild-type gilts to generate heterozygous E1 pigs. All E1 pigs were screened for the presence of the edited *CD163* allele (*CD163* ^ΔE7/+^) and the absence of off-target alterations, as described in Burger et al. (2024). The screened heterozygous E1 animals were mated within each line to generate E2 populations. The segregating E2 populations were genotyped for edited allele zygosity using the end-point TaqMan genotyping assay for the *CD163* ^ΔE7^ edit. In this study, we communicate a full set of the testing for disease resistance, phenotypical characteristics, and meat composition and quality evaluations for one maternal line. Homozygous-edited (*CD163* ^ΔE7/ΔE7^) and homozygous “null” (*CD163* ^+/+^) animals observed for phenotypic and meat composition and quality evaluations included E2 generation litters segregating for *CD163* ^ΔE7^. For disease challenge studies, E2 and E3 homozygous-edited (*CD163* ^ΔE7/ΔE7^), heterozygous-edited (*CD163* ^ΔE7/+^), and homozygous “null” (*CD163* ^+/+^) animals were utilized. Figure 1 shows the breeding approach to generate homozygous-edited E2 (and beyond) pigs.

### 2.2 Disease challenge studies

The PRRSV infection studies were conducted at the biosafety level 2 (BSL2) facilities at Midwest Veterinary Services (MVS, Inc.) in Oakland, NE. Research at this facility was conducted under FDA INAD (FDA file number: I-012878) for the continued investigational development of gene-edited domestic pigs. The pigs involved in this study were sourced from a high-health PIC farm in Kentucky and were PRRSV-negative. Pigs derived from E2 and E3 generations were matched by size and age, and weaned at approximately 21 days of age. Pigs were transported to MVS in two batches and provided non-medicated feed and water *ad libitum* throughout the study period. A similar number of pigs were assigned by genotype in each room. The PRRSV isolate assignment was randomized by room. Following 1 week of acclimation, when pigs were 28 days of age, they were intranasally inoculated with PRRSV, seeking a target dose of 10^4^–10^5^ TCID_50_. Six PRRSV isolates, with one of type 1 and five of type 2 isolates, were used for *in vivo* challenges and are identified in Table 1. The first batch of E2 and E3 pigs had three groups of pigs for separate inoculations with isolates 1-4-4 L1C, NVSL97, and SD13–15 E2. These groups had 13 homozygous and 12–13 heterozygous or null pigs (positive controls) per room. The first batch of pigs was used to comprehensively characterize PRRSV resistance and susceptibility using a PRRSV PCR and ELISA diagnostic tests, thereby measuring rectal temperatures, depression, and respiratory scores. The second batch of pigs was from an E3 generation with three groups for separate inoculations with isolates 1-8-4 L1H, 1-7-4 L1A, and 1-4-2 L1E. Because resistance is a binary trait—pigs are either positive or not—these groups had five homozygous and five null pigs (positive controls) and were used to test additional isolates, and resistance and susceptibility were only tested by PRRSV PCR and ELISA.

**TABLE 1:** PRRSV isolates used in challenge studies.

Type	Virus isolate	Background	Reference
1	SD03-15 (SD13-15)	A type 1 (EU) PRRSV isolate demonstrated to produce viremia that is present in the United States of America PRRSV type I genetic evolution has continued to diverge from PRRSV type 2	Wells et al. (2017)
2	UIL21-0712 (1-4-4 1C)	This isolate represents a lineage that emerged around 2020 and is now the most prevalent PRRSV lineage in the United States of America. It is associated with respiratory signs, abortions, and viremia	Yan et al. (2022)
2	NVSL97-7895 (NVSL97)	Emerged in the United States of America in 1996 and has been responsible for several outbreaks in the field. It is demonstrated to produce viremia and reproductive disease	Ladinig et al. (2015)
2	USA/NE/26342–1/2022 (1-8-4 L1H)	Regularly detected lineage with increased genetic diversity, common isolates from Lineage 1H. In 2023, this isolate was commonly detected in the Iowa State University Veterinary Diagnostic Laboratory (ISU VDL)	PRRSView^a^
2	USA/IN/65239-GA/2014 (1-7-4 L1A)	Lineage 1A was the most common lineage from 2015 until recently. This lineage reemerged in 2015 with the spread of the RFLP type 1-7-4 isolate. In 2023, this isolate was commonly detected at the ISU VDL	PRRSView^a^
2	USA/OK/27915–12/2022 (1-4-2 L1E)	Lineage 1E is less frequently detected than other Lineage 1 sub-lineages, but still commonly detected at the ISU VDL	PRRSView^a^

^a^ PRRSView: prrsv.vdl.iastate.edu, site visited in October 2023.

NVSL97 and SD13-15 were sourced from Dr. Raymond Rowland, Jr., University of Illinois, Department of Pathobiology; the 1-4-4 L1C isolate was sourced from Dr. Ying Fang, University of Illinois, Department of Pathobiology; and the linage 1 isolates 1-8-4 L1H, 1-7-4 L1A, and 1-4-2 L1E were sourced from Dr. Jianqiang Zhang, Veterinary Diagnostic Laboratory, Iowa State University (ISU VDL). All PRRS viruses were grown on MARC-145 cells.

Pigs inoculated with isolates 1-4-4 L1C, NVSL97, and SD13-15 received 3 mL of inoculum, and 1½ mL was administered in each nostril. Pigs inoculated with isolates 1-8-4 L1H, 1-7-4 L1A, and 1-4-2 L1E received 5 mL of inoculum or 2½ mL in each nostril. The inoculum volume of 3 or 5 mL was selected based on the advice of the virologist who provided the virus stock solutions as they had previously experienced with inoculation studies with those isolates.

Pigs from batch 1 were monitored twice daily and bled a day before the inoculation (day −1), and on days 3, 7, 10, 14, and 21 post-inoculation when the study was terminated. Sera were collected from blood samples through centrifugation, aliquoted, and stored in the −20°C freezer. Sera samples were submitted to ISU VDL to be tested with the Thermofisher VetMAX™ EU & NA 2.0 Kit for PRRSV PCR and IDEXX PRRS X3 ELISA to detect PRRSV nucleic acid and antibody seroconversion, respectively. Samples tested by PCR were considered positive for PRRSV nucleic acid detection if a cycle threshold (Ct) of less than 37 was detected and negative if Ct was ≥37. In this study, PCR results between 35.4 and 36.9 were considered a suspect. The samples tested by ELISA were considered positive for detecting PRRSV antibodies if the S/P-value was ≥0.4 and negative if the S/P value was below 0.4.

The pigs were monitored twice daily, and temperatures, depression, and respiratory scores (0–3) were recorded every morning. The model used for each strain to calculate the temperature least squares means is expressed as

$$y_{ijkl}=\mu +z_{i}+s_{j}+p_{k}+{sp}_{jk}+e_{ijkl},$$

where *y*_*ijk*_ represents the temperature (days 0–2), *µ* represents the mean, *z*_*i*_ represents the *i* ^th^ zygosity, *s*_*j*_ represents the *j* ^th^ sex effect, *p*_*k*_ represents the *k* ^th^ pen effect, *sp*_*jk*_ represents the sex × pen interaction effect, and *e*_*ijkl*_ represents the residual. The differences between zygosities were defined as statistically significant at the *p* < 0.05 level. The depression scores ranged as follows: normal: 0, within normal limits, alert, active, and normal appetite; mild: 1, moves slower, slightly rough coat, and appears lethargic, but moves around with stimulation; moderate: 2, inactive, may be recumbent but can stand, gaunt, and may be dehydrated; and severe: 3, down and unable to get up, gaunt, and dehydrated. The respiratory scores ranged as follows: 0, normal; mild: 1, slightly increased the respiratory rate and effort and nasal ocular discharge; moderate: 2, increased the respiratory rate, with some abdominal breathing; severe: 3, increased the respiratory rate with abdominal efforts, open mouth breathing, grunting, or dog sitting. Scores were recorded for pigs alive with a score per day. Mortalities were recorded.

The caregivers at the BSL2 facilities were blinded to the individual identifications and zygosities of the pigs. The ISU VDL diagnosticians testing the samples using PCR and ELISA were also blinded.

Pigs were transported following the Transport Quality Assurance (TQA) certification program from the National Pork Board, and Institutional Animal Care and Use Committee (IACUC) approvals were granted for the infection studies conducted at MVS^3^. Pigs were supervised twice a day by a veterinarian during the duration of the study.

None of the pigs involved with the inoculation studies were part of the phenotypic characterization below as they were removed from the PRRSV negative farm of origin and exposed to PRRSV.

### 2.3 Phenotypic characterization of edited pigs

Animals selected for the study were evaluated for various phenotypic characteristics corresponding to the life stage of the pigs: early life, finishing, reproduction, post-mortem, and meat composition. All animals included in these observational studies were under the supervision of staff veterinarians. Unless noted otherwise, all measures described in detail below were collected using methodologies identical to those used in the Genus PIC’s commercial genetic improvement program, allowing direct comparison with the unedited pigs of the same breeding line in the company’s overall breeding population. For purposes of this study, the “reference population” includes routinely recorded historical data from all unedited animals of the same breeding line at two of Genus PIC’s nucleus farms for the full calendar year prior to the observational studies (2021).

The pool of initial candidate animals for phenotypic characterization included piglets from multiple E1 gilt farrowings within an 18-day farrowing window, 121 homozygous-edited (*CD163* ^ΔE7/ΔE7^) and 151 homozygous-unedited (null, *CD163* ^+/+^) pigs in total. All candidate animals were measured for early life characteristics—individual piglet birth weights and the total teat count were recorded within 24 h of birth. The genotypes of the pigs were confirmed by a TaqMan^®^ assay from tissue samples collected on day 3 after birth. Upon weaning (average 21 days of age), piglets were moved to a traditional nursery, where they were randomly placed in pens of approximately 15–20 pigs, grouped by sex.

In order to create balanced study pens for the finishing stage, upon movement from nursery to finisher pens, a subset of pigs from among the larger candidate pool were randomly allocated among eight equal-sized pens based upon sex and zygosity. Four pens were allocated to each sex (boars or gilts). Each pen contained seven animals of each zygosity, homozygous-edited and homozygous null, with one minor exception: due to limitations in animal availability at the time of sorting, one boar pen held eight edited animals and six nulls, while one gilt pen held six edited animals and eight nulls. In total, the finishing-stage observations included 55 animals of each genotype. Animals from the earlier nursery stage that were not needed for the finishing study were not evaluated further. During the finishing period, pigs had *ad libitum* access to feed and water. At approximately 140 days of age, body weight and ultrasound measurements of backfat and loin depth were recorded on each pig. Backfat and loin depth were measured with an ExaGo^4^ ultrasound machine, and estimates were retrieved using BioSoft Toolbox^5^ software. The lifetime daily gain was calculated by dividing the 140-day weight by the actual age of the pig on the weight date.

At approximately 180 days of age, pigs were allocated to either stay on-site for reproductive characterization or sent off-site for the assessment of meat quality traits and compositional analysis (described further below). The remaining gilts were grown to sexual maturity and were characterized for their reproductive traits until their progeny were born. Gilts were observed for a first and second estrus, and then were mated and allowed to farrow their first litter. The first six gilts to farrow after mating were used for analysis. Within 24 h of farrowing, the total number of piglets born, the number of piglets born alive, the number of piglets born dead, and the number of mummified piglets were recorded for each litter. Gestation length was also computed as the difference between the farrowing date and mating date.

The carcasses and organs of animals selected for the evaluation of meat characteristics were visually evaluated by a Genus PIC veterinarian for various physiological defects, including skin lesions, nasal turbinate lesions, percent of lung lesion involvement, pericarditis, pleuritis, peritonitis, liver lesions, and the presence of gastric ulcers (Christensen and Cullinane, 1990; PigMON Slaughter Inspection Procedures Manual, 1992; Robertson et al., 2002; Steinmann et al., 2014; Bottacini et al., 2018).

### 2.4 Statistical analysis of phenotypic data

Statistical analyses were carried out in SAS (SAS Institute Inc., Cary, NC, United States of America) using PROC GLM to estimate least-squared means, standard errors, and tests for significance. For all tests, differences between zygosities were defined as statistically significant at the *p* < 0.05 level. The *p*-values reported herein did not take into account the multiple comparison correction across traits. The statistical model used for birth weight and teat count is expressed as

$$y_{ijk}=\mu +z_{i}+s_{j}+e_{ijk},$$

where *y*_*ijk*_ represents the record, *µ* represents the mean, *z*_*i*_ represents the *i* ^th^ zygosity, *s*_*j*_ represents the *j* ^th^ sex effect, and *e*_*ijk*_ represents the residual. For the traits recorded at the end of the finishing stage, pigs were intentionally placed in pens balanced for zygosity. Because pens were constrained to a single gender, pens were nested within a gender. Accordingly, the model for 140-day weight and leg scores is expressed as

$$y_{ijklm}={\mu +z}_{i}+s_{j}+g_{k}+p_{l\left(j\right)}+e_{ijklm},$$

where *y*_*ijklm*_ represents the record, *µ* represents the mean, *z*_*i*_ represents the *i* ^th^ zygosity, *s*_*j*_ represents the *j* ^th^ sex effect, *g*_*k*_ represents the *k* ^th^ group effect, *p*_*l(j)*_ represents the *l* ^th^ pen nested with the *j* ^th^ sex, and *e*_*ijklm*_ represents the residual. As loin depth and backfat can be affected by the size of the animal, the statistical model for loin depth and backfat is expressed as

$$y_{ijklm}={\mu +z}_{i}+s_{j}+g_{k}+p_{l\left(j\right)}+w_{ijklm}\beta +e_{ijklm},$$

where *y*_*ijklm*_ represents the record, *µ* represents the mean, *z*_*i*_ represents the *i* ^th^ zygosity, *s*_*j*_ represents the *j* ^th^ sex effect, *g*_*k*_ represents the *k* ^th^ group effect, *p*_*l(j)*_ represents the *l* ^th^ pen nested with the *j* ^th^ sex, *w*_*ijklm*_ *β* represents the 140-d weight covariate for the *ijklm* ^th^ individual, and *e*_*ijklm*_ represents the residual. The statistical models for the evaluation of gestation length, total number of piglets born, number of piglets born alive, number of piglets born dead, and number of mummified pigs can be expressed as

$$y_{ij}={\mu +z}_{i}+e_{ij},$$

where *y*_*ij*_ represents the record, *µ* represents the mean, *z*_*i*_ represents the *i* ^th^ zygosity, and *e*_*ij*_ represents the residual. The statistical model for the post-mortem evaluation of physiological organ traits is expressed as

$$y_{ijnl}={\mu +z}_{i}+s_{j}+{h_{n}+e}_{ijnl},$$

where *y*_*ijnl*_ represents the record, *µ* represents the mean, *z*_*i*_ represents the *i* ^th^ zygosity, *s*_*j*_ represents the *j* ^th^ sex effect, *h*_*n*_ represents the *n* ^th^ harvest date effect, and *e*_*ijnl*_ represents the residual. As pens were not part of the trial design for the post-mortem traits, pens were not used in the statistical model.

### 2.5 Meat quality traits and compositional analysis of edible tissues

At approximately 205 days in age, 20 animals (10 boars and 10 gilts) of each genotype, homozygous-edited and homozygous null, were selected from among the animals in the finishing stage of the phenotypic observational studies described above. To minimize the impact of disparities in the pigs' growth rate, a group of pigs with an age variation of fewer than 10 days and a weight variation of less than 25 kg was used in the study. The selected pigs were then shipped to the Purdue University meat processing laboratory for carcass preparation, evaluation, and collection of tissue samples. Standard industry practices were used for pig harvesting and for carcass-chilling conditions (Harris et al., 2017; Boler, 2019; Matthews et al., 2022) and were overseen by inspectors from the Indiana Board of Animal Health and Genus PIC veterinarians.

Within approximately 30 min of stunning, hot carcass weight was recorded. Subsequently, each half of the carcass was moved into a cooler and set at approximately 2°C, for approximately 24 h of chilling. Twenty-four hours after post-mortem, 10 out of 12 carcasses from each genotype (five boars and five gilts each) were randomly selected for further characterization, including backfat thickness (the last and 10^th^ rib position), subjective and objective color, and subjective marbling. Four samples, each weighing approximately 400–500 g, were cut from each longissimus dorsi muscle, and trimmed of visible fat and connective tissue. Each sample was then sliced into four pieces longitudinally to muscle direction, frozen in liquid nitrogen, and stored in a −80°C freezer.

Two of the four frozen loin muscle samples were randomly selected from each carcass and were shipped to Eurofins Food Chemistry Testing Inc. in Madison, WI, for compositional analysis. At Eurofins, loin samples were ground in liquid nitrogen, and the edible tissue composition variables were analyzed, following standardized Eurofins methodologies. All animals, carcasses, and meat samples were given randomly assigned identifiers such that Purdue University and Eurofins staff were, at all times, blind to the sample genotypes.

The statistical analyses of meat characteristics and compositional analysis were carried out in SAS software using the PROC GLM function to estimate least-squared means, standard errors, and tests for significance at *p* < 0.05. For carcass and raw meat traits, the model effects were zygosity, sex, hot carcass weight, and the sample collection day. The sex effect and collection day were fitted as a class fixed effect, whereas hot carcass weight was used as a linear covariate in the analysis. For Eurofins evaluated traits, the model effects were zygosity, sex, and collection date. These three effects were all fitted as class fixed effects. The least-squared means and pairwise *p*-value were provided for zygosity and sex. For variable characterization, means, mean differences, and standard deviation values were calculated to determine substantial equivalence between zygosities.

## 3 Results

### 3.1 Disease challenge studies

Overall, the challenge inoculum for isolates 1-4-4 L1C and 1-7-4 L1A was on target (within the 10^4^–10^5^ TCID_50_ range), but some isolates, including NVSL97 and SD13-15, were below the desired target dose, and isolates 1-8-4 L1H and 1-4-2 L1E were above the target (Table 2).

**TABLE 2:** PRRSV concentration of the stock solution and final challenge material.

PRRSV isolate	PRRSV concentration in the challenge inoculum (mL)
1-4-4 L1C	4.64 × 10^3^ TCID_50_/mL = 1.4 × 10^4^/3
NVSL97	3.16 × 10^3^ TCID_50_/mL = 9.5 × 10^3^/3
SD13-15	2.15 × 10^3^ TCID^50^/mL = 6.5 × 10^3^/3
1-7-4 L1A	1.78 × 10^5^ TCID_50_/mL = 8.9 × 10^5^/5
1-8-4 L1H	6.81 × 10^5^ TCID_50_/mL = 3.4 × 10^6^/5
1-4-2 L1E	3.16 × 10^5^ TCID_50_/mL = 1.6 × 10^6^/5

All pigs were PCR- and ELISA-negative at arrival to the BSL2 facilities, demonstrating that they were naïve before the inoculation. They remained negative during the acclimation period, and no cross-contamination occurred between rooms. The homozygous-edited pigs were negative for PRRSV by PCR, across the bleeding times and up to 21 days post-inoculation. This was the case for pigs challenged with all isolates tested, including 1-4-4 L1C, NVSL97, SD13-15, 1-7-4 L1A, 1-8-4 L1H, and 1-4-2 L1E. Two homozygous pigs challenged with 1-4-4 L1C (pigs 252 and 835) and one challenged with 1-8-4-L1H (pig 835) had single PCR-positive results on day 7; however, they tested PCR-negative at the next bleeding period and stayed negative for the remainder of the study. In contrast, the heterozygous and null pigs were PCR-positive when challenged with the same isolates.

None of the homozygous-edited pigs mounted an immune response to PRRSV detectable by ELISA. In contrast, most heterozygous and null pigs inoculated with isolates seroconverted. Only a few null pigs did not seroconvert by a 21-day post-challenge, including pig 371 inoculated with NVSL97; pigs 281, 357, 359, 365, and 400 inoculated with SD13-15; and pig 170 inoculated with the isolate 1-7-4. A null pig (824) inoculated with the isolate 1-4-2 L1E showed seroconversion at days 10 and 14 and became serologically negative at day 21.

The summaries of PCR and ELISA data are shown in Tables 3, 4, and individual pig PRRSV PCR and serology results are shown in Supplementary Tables S1–S6. The rectal temperatures in homozygous pigs were statistically lower than those of heterozygous or null pigs when challenged with the highly virulent isolate 1-4-4 L1C on days 3–5, 7–10, 12–13, and 20. These differences were less evident in pigs challenged with the isolate NVSL97, where homozygous pigs had significantly lower temperatures on days 10, 15, and 20. In pigs challenged with the less-virulent isolate SD13-15, homozygous pigs had lower temperatures than null pigs only on day 17 (Supplementary Figure S1).

**TABLE 3:** Summary of PCR results by zygosity and isolate. Results are expressed as the proportion of pigs that were positive by PCR before inoculation (day 1) and post-inoculation (days 3–21). PCR Ct values <37 are considered positive. HOM, homozygous-edited (*CD163* ^ΔE7/ΔE7^); HET, heterozygous (*CD163* ^ΔE7/+^); NULL, unedited null segregants (*CD163* ^+/+^).

		% Positive PCR (Ct < 37)					
		Days post-inoculation					
Isolate	Zygosity	−1	3	7	10	14	21
1-4-4 L1C	HOM (n = 13)	0	0	7.7	0	0	0
	HET (n = 8)	0	100	100	100	100	100
	NULL (n = 4)	0	100	100	100	100	100^a^
NVSL97	HOM (n = 13)	0	0	0	0	0	0
	HET (n = 7)	0	86	100	100	100	100
	NULL (n = 5)	0	100	100	100	100	100
SD13-15	HOM (n = 12)	0	0	0	0	0	0
	HET (n = 8)	0	50	63	75	75	100^a^
	NULL (n = 5)	0	0	0	20	80	100
1-8-4 L1H	HOM (n = 5)	0	0	0	0	0	0
	NULL (n = 5)	0	100	100	100	100	100
1-7-4 L1A	HOM (n = 5)	0	0	0	0	0	0
	NULL (n = 5)	0	100	100	100	100	100
1-4-2 L1E	HOM (n = 5)	0	0	0	0	0	0
	NULL (n = 5)	0	100	100	100	100	100

^a^ This group contains animals that were euthanized or died during the study.

**TABLE 4:** Summary of ELISA results by zygosity and isolate. Results are expressed as the proportion of pigs that were positive by ELISA before inoculation (day 1) and post-inoculation (days 3–21). ELISA S/P ratios equal or higher than 0.4 are considered positive. HOM, homozygous edited (*CD163* ^ΔE7/ΔE7^); HET, heterozygous (*CD163* ^ΔE7/+^); NULL, unedited null segregants (*CD163* ^+/+^).

		% Positive ELISA (S/P ≥ 0.4)					
		Days post-inoculation					
Isolate	Zygosity	−1	3	7	10	14	21
1-4-4 L1C	HOM (n = 13)	0	0	0	0	0	0
	HET (n = 8)	0	0	12.5	100	100	100
	NULL (n = 4)	0	0	0	100	100	100^a^
NVSL97	HOM (n = 13)	0	0	0	0	0	0
	HET (n = 7)	0	0	0	14.2	86	86
	NULL (n = 5)	0	0	0	57	100	100
SD13-15	HOM (n = 12)	0	0	0	0	0	0
	HET (n = 8)	0	0	0	14.2	57.1	57.1
	NULL (n = 5)	0	0	0	0	0	40^a^
1-8-4 L1H	HOM (n = 5)	0	0	0	0	0	0
	NULL (n = 5)	0	0	0	80	100	100
1-7-4 L1A	HOM (n = 5)	0	0	0	0	0	0
	NULL (n = 5)	0	0	20	80	40	60
1-4-2 L1E	HOM (n = 5)	0	0	0	0	0	0
	NULL (n = 5)	0	0	0	100	100	80

^a^ This group contains animals that were euthanized or died during the study.

Only depression and respiratory scores of 0 and 1 were observed throughout the study, while scores of 2 and 3 were not observed, regardless of zygosity. The results showed that the proportion of depression scores of 1 was always lower in homozygous pigs than those in heterozygous and null pigs. Additionally, the proportions of respiratory scores were almost always lower in homozygous pigs than those in heterozygous and null pigs, with the single exception of respiratory scores in null pigs challenged with NVSL97 (Supplementary Table S7). The lower proportions of depression and respiratory scores were observed across inoculations with 1-4-4 L1C, NVSL97, and SD13-15.

The mortality in this study was low, and only two pigs died; null pig 287 inoculated with the isolate 1-4-4 L1C was lame and had to be humanely euthanized, and null pig 288 inoculated with SD13-15 was found dead. No necropsies were performed.

### 3.2 Phenotypic characterization of edited pigs

Table 5 summarizes key phenotypic characteristics observed across the lifecycle of the pigs. Across all characteristics recorded, no significant differences were identified between homozygous-edited (*CD163* ^ΔE7/ΔE7^) and homozygous-null (*CD163* ^+/+^) animals, with a single exception discussed further below. Aside from this exception, no differences were observed in early life (birthweight and teat number), finishing stage (weight, weight gain, and loin depth), or the female reproductive capacity (the gestational period and number of piglets born, alive, or dead/mummified).

**TABLE 5:** Least-squared mean tests of significance (including the standard error of the mean, SEM) between homozygous-edited (*CD163* ^ΔE7/ΔE7^) and homozygous-unedited (null, *CD163* ^+/+^) pigs for phenotypic characteristics across the lifecycle of the pigs. Descriptive statistics from non-edited animals of the same breeding line from two of genus PIC’s nucleus farms in 2021 are also included for comparison (the “reference population”).

	Pigs in observational studies				Reference population^a^					
	*CD163* ^ΔE7/ΔE7^		*CD163* ^+/+^		—					
Trait^b^	N	LS mean (SEM)	N	LS mean (SEM)	N	Mean	SD	Min	Max	
Early life traits										
BWT	121	1.30 (0.03)	138	1.26 (0.03)	30,464	1.39	0.34	0.20	2.70	
TEAT	121	15.91 (0.12)	138	15.97 (0.12)	12,062	15.69	1.25	6	20	
Finishing traits										
WT140	55	93.17 (1.30)	55	88.77 (1.30)	12,064	97.07	9.74	56.00	136.50	
LDG	55	636.96 (8.29)	55	611.83 (8.29)	12,064	685.65	68.28	397.00	942	
BF	55	8.98 (0.23)^c^	55	9.99 (0.23)^c^	12,066	8.48	2.23	3.3	22.7	
LD	55	63.54 (0.65)	55	65.09 (0.64)	12,064	59.66	5.93	34.4	93.4	
Female reproductive traits										
GL	6	116.2 (0.4)	6	116.8 (0.4)	992	116.6	1.5	112		123
TNB	6	12.7 (1.1)	6	13.3 (1.1)	992	15.6	3.4	1		25
NBA	6	11.8 (1.1)	6	13.0 (1.1)	992	14.2	3.2	0		23
NBD	6	0.8 (0.4)	6	0.3 (0.4)	992	1.4	1.6	0		11
MUM	6	0.2 (0.1)	6	0.0 (0.1)	992	0.5	0.9	0		8

^a^ The “reference population” includes all historical data collected from data from the non-edited animals of the same breeding line at two of the Genus PIC’s nucleus farms in calendar year 2021.

^b^ BWT, individual piglet birth weight, kg; TEAT, total teat count; WT140, individual weight of pig at 140 days of age, kg; LDG, lifetime daily gain, g per day; BF, ultrasound predicted 10th rib backfat depth, mm; LD, ultrasound predicted 10th rib loin depth, mm; GL, gestation length, days; TNB, total number of piglets born; NBA, number of piglets born alive; NBD, number of piglets born dead ; MUM, number of mummified piglets.

^c^ *p* < 0.05. Not significant after Bonferroni multiple-test correction.

The sole exception among the traits measured was a statistically significant difference between the two genotypes in ultrasound-predicted 10^th^ rib backfat depth. Although the means for both genotypes fell within the normal range of the reference population, null animals in the study appeared to have a greater backfat depth than the homozygous-edited animals or the mean of the reference population. This observation is inconsistent with meat compositional analysis (see below), where no significant differences in meat weight, marbling, or fat composition between the two genotypes were observed. Additionally, the phenotypic results reported here do not include Bonferroni correction for multiple tests; when applied, this observation is insignificant. Regardless, the unusual observation does not appear to be associated with the presence of *CD163* ^ΔE7^.

### 3.3 Meat quality traits and compositional analysis of edible tissues

Table 6 summarizes the meat quality traits and compositional data collected at the Purdue University meat processing laboratory and Eurofins Food Chemistry Testing, respectively. Across all characteristics recorded, no significant differences were identified between homozygous-edited (*CD163* ^ΔE7/ΔE7^) and homozygous-null (*CD163* ^+/+^) animals. The values for all measured variables fall within the normal range, as described in the reference publications (Huff-Lonergan et al., 2002; Huff-Lonergan and Page, 2002; Harris et al., 2017; Boler, 2019; Matthews et al., 2022). Thus, we find no evidence to suggest that *CD163* ^ΔE7^ has any adverse impact on meat quality or composition. Furthermore, no significant differences were observed in post-mortem physical carcass defects (also included in Table 6).

**TABLE 6:** Least-squared mean tests of significance (including the standard error of the mean, SEM) between homozygous-edited (*CD163* ^ΔE7/ΔE7^) and homozygous-unedited (null, *CD163* ^+/+^) pigs for various meat quality measures, compositional analysis of edible tissues, and carcass defects (N = 10 animals per genotype, including five gilts and five boars each). None of the comparisons were significantly different at the *p* < 0.05 level.

	*CD163* ^ΔE7/ΔE7^	*CD163* ^+/+^
Meat quality measures		
Hot carcass weight, 30 min post-mortem (kg)	99.7 (1.86)	104.5 (1.86)
Longissimus muscle pH, 24 h post-mortem	5.80 (0.02)	5.79 (0.03)
NPB color score (1–5)^a^	3.1 (0.10)	2.9 (0.10)
NPB marbling score (1–10)^b^	1.4 (0.10)	1.3 (0.11)
Hunter color L (lightness)^c^	38.1 (0.59)	37.5 (0.60)
Hunter color a (redness)^c^	7.2 (0.23)	7.5 (0.23)
Hunter color b (yellowness)^c^	3.5 (0.19)	3.6 (0.20)
Meat compositional analysis		
Protein, %	23.1 (0.10)	23.0 (0.10)
Moisture, %	75.7 (0.11)	75.9 (0.11)
Ash, %	0.99 (0.01)	1.03 (0.01)
Carbohydrate, %	LOQ^d^	LOQ^d^
Total fat, %	1.14 (0.07)	1.03 (0.07)
Total saturated fatty acids, %	0.360 (0.0297)	0.321 (0.0291)
Total monounsaturated fatty acids, %	0.0639 (0.0034)	0.0601 (0.0033)
Total polyunsaturated fatty acids, %	0.215 (0.0044)	0.210 (0.0043)
Calories, Kcal/100g	103 (0.76)	101 (0.74)
Cholesterol, mg/100g	45.7 (0.49)	45.5 (0.48)
Vitamin B12 (cobalamin), mcg/g	0.00475 (0.0003)	0.00371 (0.0003)
Vitamin B3 (niacin), mcg/g	108 (3.58)	104 (3.50)
Vitamin B6 (pyridoxine HCl),mcg/g	9.39 (0.26)	9.72 (0.25)
Vitamin E (alpha tocopherol), mcg/g	3.14 (0.14)	3.06 (0.14)
Iron, ppm	4.00 (0.09)	3.86 (0.09)
Carcass defects		
Skin lesion, 0–2^e^	0	0
Nasal turbinate lesion, 0–5	0.0 (0.04)	0.1 (0.04)
Lung lesion percent, 0–100	1.7 (0.47)	0.8 (0.47)
Pericarditis, 0/1^e^	0	0
Pleuritis, 0/1^e^	0	0
Peritonitis, 0/1^e^	0	0
Liver lesion, 0–2^e^	0	0
Gastric ulcer, 0–3	0.2 (0.15)	0.1 (0.15)

^a^ National Pork Board Pork color score (1 = lightest and 5 = darkest).

^b^ National Pork Board Pork marbling score (1 = devoid of fat and 10 = abundant marbling).

^c^ https://support.hunterlab.com/hc/en-us/articles/204137825-Measuring-Color-using-Hunter-L-a-b-versus-CIE-1976-L-a-b-AN-1005b

^d^ Below limit of quantitation.

^e^ All observations were 0.

## 4 Discussion

The described studies aimed to evaluate if the removal of the SRCR5 domain from CD163 protein confers resistance to PRRSV isolates and has any unexpected effect on pigs’ phenotypical characteristics or changes in meat quality and composition.

The results of the disease challenge studies confirmed that the homozygous-edited pigs were resistant to PRRSV when challenged intranasally with the North American PRRSV type 2 isolates 1-4-4 L1C, NVSL97, 1-8-4 L1H, 1-7-4 L1A, and 1-4-2 L1E and the type 1 isolate SD13-05, as demonstrated by the negative PCR and ELISA results up to 21 days post-infection. In contrast, as expected, heterozygous and null pigs were positive. Homozygous pigs did not mount an immune response as the immune system did not recognize PRRSV due to the lack of cellular infection. The timing of PCR and ELISA positive responses in heterozygous and null pigs varied according to the virulence of the isolate with 1-4-4 L1C, 1-8-4 L1H, 1-7-4 L1A, and 1-4-2 L1E, resulting in 100% of heterozygous and null pigs becoming PCR-positive by day 3, followed by 92% of NVSL97-inoculated pigs. The least virulent isolate was the PRRSV type 1 SD13-15, and it took 21 days for all heterozygous and null pigs to become PCR-positive. Resistance to PRRSV was evident in homozygous-edited pigs from E2 and E3 generations to all isolates that emerged in more than the last two decades, with the oldest isolate originating in 1996 (NVSL97) and the most recent being 2022 (1-8-4 L1H and 1-4-2 L1E).

Two homozygous pigs showed positive PCR titers ranging from 35.4 (1-8-4 L1H) to 35.7 (1-4-4 L1C) at a single timepoint. In this study, Ct ranges between 35.4 and 36.9 were suspected as false-negative. It is possible that those pigs were positive transiently, following the inoculation, or were infected from their environment as they were housed in the same room as heterozygous and null pigs that were PCR-positive and likely shedding the virus. Nevertheless, they became PCR-negative at the next testing period (within 3 days) and never mounted an immune response.

A few heterozygous and null pigs inoculated with the SD13-15 isolate, one pig inoculated with NVSL97, and another one with the 1-7-4 L1A isolate did not seroconvert. The result observed for the SD13-15 isolate is unsurprising as this type 1 isolate is considered a low-virulence isolate; several of the pigs that did not seroconvert became PCR-positive by day 21. However, it was surprising that one pig inoculated with an NVSL97 isolate and another one with 1-7-4 L1A did not seroconvert, especially considering that they became PCR-positive by day 3 and stayed positive until day 21. The reasons why the immune system did not mount an immune response by then were unclear.

Under the conditions of these studies, neither homozygous nor heterozygous or null pigs inoculated with PRRSV showed the acute clinical signs typically observed in commercial pigs and had overall low depression and respiratory scores (1). This may be explained by the fact that these pigs were sourced from a high-health farm and managed with minimal stress, which differs from disease expression under commercial conditions. Nevertheless, homozygous pigs showed a lower proportion of depression and respiratory scores of 1 than in heterozygous and null pigs, supporting the PCR and ELISA data.

The type 2 PRRSV isolates used for disease challenges represent the most dominant contemporary lineages and sub-lineages, according to the University of Minnesota Veterinary Diagnostics Laboratory (UMN VDL), and the ISU VDL is the USA’s leading reference VDLs for swine disease. In 2022, based on ORF5 sequence analysis, approximately 41% of all UMN VDL sequences were L1C, and 1/3 comprised the L1C 1-4-4 variant. The second most common lineage/sub-lineage was L1H (24%), followed by L1A (14%) and L1E (11%). Non-L1 sequences (L2 to L9) represented 8% of all sequences [personal communication from Dr. Albert Rovira, Interim Director. UMN VDL, updating the data earlier published by Paploski et al. (2021)]. Considering these frequencies, this study included isolates from at least 90% of the most dominant and contemporary PRRSV lineages and sub-lineages. Like UMN VDL, ISU VDL also reported similar PRRSV lineages and sub-lineage frequencies^6^.

The results of phenotypical characterization support the conclusion that *CD163* ^ΔE7^ has no observable impact on the pigs’ health, growth, and productivity. The edited animals behaved, grew, and reproduced undistinguishable from the non-edited animals. Furthermore, the meat quality and composition obtained from edited pigs were the same as those obtained from non-edited pigs, further confirming that *CD163* ^ΔE7^ does not exhibit an unexpected impact. Based on all the described observations, we conclude that pigs with the SRCR5 domain removed from CD163 protein are not different from the control pigs, except for the resistance to the infection caused by the PRRS virus.

## Data Availability

The original contributions presented in the study are included in the article/Supplementary Materials; further inquiries can be directed to the corresponding author.

## Footnotes

1 PRRS cumulative incidence. Morrison Swine Health Monitoring Program. Retrieved 2 May 2023, from https://mshmp.umn.edu/reports#Charts

2 All research conducted in this report was subject to authorization and oversight by the U.S. Food and Drug Administration (INAD I-012878) for the investigational development of pigs containing the intentional genomic alteration (IGA) of the *CD163* gene. The claims of safety and efficacy of the IGA are currently under review by FDA.

3 Study MCL 23024: IACUC #AC23034P; study MVS 23038: IACUC #MAC23038P.

4
https://www.imv-imaging.com/media/8522/exago-ultrasound-scanner-manual.pdf

5
https://www.biotronics-inc.com/live-animal-software

6 PRRSView: prrsv.vdl.iastate.edu, site visited in October 2023.
