# Supplementary material for: Pigs lacking the SRCR5 domain of CD163 protein demonstrate heritable resistance to the PRRS virus and no changes in animal performance from birth to maturity
Source: Front Genome Ed. 2024 Mar 13;6:1322012. doi: 10.3389/fgeed.2024.1322012 (PMC10965679; doi:10.3389/fgeed.2024.1322012)
Supplement: Supplementary file 1 [file DataSheet1.pdf]

Supplementary Material

Table S1. Summary of PCR and ELISA results by zygosity in a room with pigs inoculated with PRRSV 1-4-4 L1C. Results are expressed as individual values before inoculation (day -1) and post-inoculation (days 3-21). PCR Ct values < 37 and ELISA S/P ratios ≥ 0.4 are positive. HOM = homozygous edited ($CD163^{\Delta E7\,/\,\Delta E7}$), HET = heterozygous ($CD163^{\Delta E7\,/\,+}$), and NULL = unedited null segregants ($CD163^{+\,/\,+}$). Darker shaded areas show positive PCR and ELISA results, and lighter shaded areas show "suspect" results that can't be confidently confirmed positive. One pig had to be euthanized due to lameness, following the onsite veterinarian recommendation.

PIG ID	ZYGOSITY	PCR (Ct < 37) Days Post Inoculation						ELISA (S/P ≥ 0.4) Days Post Inoculation					
		**0**	**3**	**7**	**10**	**14**	**21**	**0**	**3**	**7**	**10**	**14**	**21**
242	HOM	≥37	≥37	≥37	≥37	≥37	≥37	<0.4	<0.4	<0.4	<0.4	<0.4	<0.4
252	HOM	≥37	≥37	35.7	≥37	≥37	≥37	<0.4	<0.4	<0.4	<0.4	<0.4	<0.4
261	HOM	≥37	≥37	≥37	≥37	≥37	≥37	<0.4	<0.4	<0.4	<0.4	<0.4	<0.4
280	HOM	≥37	≥37	≥37	≥37	≥37	≥37	<0.4	<0.4	<0.4	<0.4	<0.4	<0.4
296	HOM	≥37	≥37	≥37	≥37	≥37	≥37	<0.4	<0.4	<0.4	<0.4	<0.4	<0.4
325	HOM	≥37	≥37	≥37	≥37	≥37	≥37	<0.4	<0.4	<0.4	<0.4	<0.4	<0.4
346	HOM	≥37	≥37	≥37	≥37	≥37	≥37	<0.4	<0.4	<0.4	<0.4	<0.4	<0.4
369	HOM	≥37	≥37	≥37	≥37	≥37	≥37	<0.4	<0.4	<0.4	<0.4	<0.4	<0.4
379	HOM	≥37	≥37	≥37	≥37	≥37	≥37	<0.4	<0.4	<0.4	<0.4	<0.4	<0.4
385	HOM	≥37	≥37	≥37	≥37	≥37	≥37	<0.4	<0.4	<0.4	<0.4	<0.4	<0.4
401	HOM	≥37	≥37	≥37	≥37	≥37	≥37	<0.4	<0.4	<0.4	<0.4	<0.4	<0.4
436	HOM	≥37	≥37	≥37	≥37	≥37	≥37	<0.4	<0.4	<0.4	<0.4	<0.4	<0.4
441	HOM	≥37	≥37	≥37	≥37	≥37	≥37	<0.4	<0.4	<0.4	<0.4	<0.4	<0.4
266	HET	≥37	20.1	17.8	17.3	16.9	23.8	<0.4	<0.4	<0.4	0.73	1.07	1.26

270	HET	≥37	18.9	15.6	16.3	20.7	24.4	<0.4	<0.4	0.47	1.34	1.19	1.04
322	HET	≥37	22.2	18.7	17.2	23.7	26.0	<0.4	<0.4	<0.4	0.64	0.75	0.59
364	HET	≥37	19	17.1	19.5	21.5	22.2	<0.4	<0.4	<0.4	0.94	0.88	0.92
375	HET	≥37	16.6	16.7	19.0	23.2	26.1	<0.4	<0.4	<0.4	0.59	1.09	1.2
386	HET	≥37	19.6	18.4	20.4	24.9	28.1	<0.4	<0.4	<0.4	1.2	1.26	1.4
399	HET	≥37	16.6	15.2	14.9	19.5	25.9	<0.4	<0.4	<0.4	0.71	0.78	0.73
410	HET	≥37	14.7	15.3	17.1	19.6	30.1	<0.4	<0.4	<0.4	0.44	0.62	0.59
326	NULL	≥37	18.3	17.4	19.0	28.3	25.6	<0.4	<0.4	<0.4	1.23	1.18	1
354	NULL	≥37	20.1	19.7	19.4	23.0	24.6	<0.4	<0.4	<0.4	1.01	1.28	1.08
278	NULL	≥37	19.4	16.4	17.8	18.8	27.3	<0.4	<0.4	<0.4	1.05	1.42	1.4
287	NULL	≥37	18.9	16.2	18.3	20.4	Euthan-ized	<0.4	<0.4	<0.4	0.9	1.22	Euthan-ized

Table S2. Summary of PCR and ELISA results by zygosity in a room with pigs inoculated with PRRSV NVSL97. Results are expressed as individual values before inoculation (day -1) and post-inoculation (days 3-21). PCR Ct values < 37 and ELISA S/P ratios ≥ 0.4 are positive. HOM = homozygous edited (*CD163*$^{\Delta E7 / \Delta E7}$), HET = heterozygous (*CD163*$^{\Delta E7 / +}$), and NULL = unedited null segregants (*CD163*$^{+ / +}$). Darker shaded areas show positive PCR and ELISA results.

Pig ID	Zygosity	PCR (Ct < 37) Days Post Inoculation						ELISA (S/P ≥ 0.4) Days Post Inoculation					
		0	3	7	10	14	21	0	3	7	10	14	21
248	HOM	≥37	≥37	≥37	≥37	≥37	≥37	<0.4	<0.4	<0.4	<0.4	<0.4	<0.4
254	HOM	≥37	≥37	≥37	≥37	≥37	≥37	<0.4	<0.4	<0.4	<0.4	<0.4	<0.4
271	HOM	≥37	≥37	≥37	≥37	≥37	≥37	<0.4	<0.4	<0.4	<0.4	<0.4	<0.4
291	HOM	≥37	≥37	≥37	≥37	≥37	≥37	<0.4	<0.4	<0.4	<0.4	<0.4	<0.4
303	HOM	≥37	≥37	≥37	≥37	≥37	≥37	<0.4	<0.4	<0.4	<0.4	<0.4	<0.4
358	HOM	≥37	≥37	≥37	≥37	≥37	≥37	<0.4	<0.4	<0.4	<0.4	<0.4	<0.4
366	HOM	≥37	≥37	≥37	≥37	≥37	≥37	<0.4	<0.4	<0.4	<0.4	<0.4	<0.4
381	HOM	≥37	≥37	≥37	≥37	≥37	≥37	<0.4	<0.4	<0.4	<0.4	<0.4	<0.4
391	HOM	≥37	≥37	≥37	≥37	≥37	≥37	<0.4	<0.4	<0.4	<0.4	<0.4	<0.4
433	HOM	≥37	≥37	≥37	≥37	≥37	≥37	<0.4	<0.4	<0.4	<0.4	<0.4	<0.4
439	HOM	≥37	≥37	≥37	≥37	≥37	≥37	<0.4	<0.4	<0.4	<0.4	<0.4	<0.4
443	HOM	≥37	≥37	≥37	≥37	≥37	≥37	<0.4	<0.4	<0.4	<0.4	<0.4	<0.4
471	HOM	≥37	≥37	≥37	≥37	≥37	≥37	<0.4	<0.4	<0.4	<0.4	<0.4	<0.4
268	HET	≥37	21.3	18.5	18.2	20.4	25.4	<0.4	<0.4	<0.4	0.487	0.920	0.903
274	HET	≥37	30.4	23.0	19.0	16.0	17.9	<0.4	<0.4	<0.4	<0.4	0.616	0.905
371	HET	≥37	24.6	20.2	18.8	18.7	18.4	<0.4	<0.4	<0.4	<0.4	<0.4	<0.4
376	HET	≥37	20.8	20.3	22.3	27.1	24.1	<0.4	<0.4	<0.4	<0.4	0.494	0.527

382	HET	≥37	≥37	30.6	26.7	24.8	28.3	<0.4	<0.4	<0.4	<0.4	0.459	1.128
390	HET	≥37	23.9	20.3	17.2	22.6	18.4	<0.4	<0.4	<0.4	<0.4	0.913	0.731
403	HET	≥37	18.5	16.1	14.9	18.2	24.6	<0.4	<0.4	<0.4	<0.4	0.723	0.720
394	NULL	≥37	25.7	21.8	18.1	20.6	25.7	<0.4	<0.4	<0.4	0.597	1.115	1.075
283	NULL	≥37	22.3	19.2	16.2	21.5	26.8	<0.4	<0.4	<0.4	0.432	1.104	1.117
295	NULL	≥37	21.5	18.6	16.7	24.4	26.2	<0.4	<0.4	<0.4	<0.4	0.760	0.757
351	NULL	≥37	23.2	18.0	21.1	28.4	29.4	<0.4	<0.4	<0.4	0.609	0.942	0.834
367	NULL	≥37	22	19.5	18.9	20.5	26.9	<0.4	<0.4	<0.4	0.489	0.802	0.876

Table S3. Summary of PCR and ELISA results by zygosity in a room with pigs inoculated with PRRSV SD13-15. Results are expressed as individual values before inoculation (day -1) and post-inoculation (days 3-21). PCR Ct values < 37 and ELISA S/P ratios ≥ 0.4 are positive. HOM = homozygous edited (*CD163*$^{\Delta E7 / \Delta E7}$), HET = heterozygous (*CD163*$^{\Delta E7 / +}$), and NULL = unedited null segregants (*CD163*$^{+ / +}$). Darker shaded areas show positive PCR and ELISA results. One pig in this group was found dead.

Pig ID	Zygosity	PCR (Ct < 37) Days Post-inoculation						ELISA (S/P ≥ 0.4) Days Post-inoculation					
		**-1**	**3**	**7**	**10**	**14**	**21**	**0**	**3**	**7**	**10**	**14**	**21**
244	HOM	≥37	≥37	≥37	≥37	≥37	≥37	<0.4	<0.4	<0.4	<0.4	<0.4	<0.4
253	HOM	≥37	≥37	≥37	≥37	≥37	≥37	<0.4	<0.4	<0.4	<0.4	<0.4	<0.4
262	HOM	≥37	≥37	≥37	≥37	≥37	≥37	<0.4	<0.4	<0.4	<0.4	<0.4	<0.4
299	HOM	≥37	≥37	≥37	≥37	≥37	≥37	<0.4	<0.4	<0.4	<0.4	<0.4	<0.4
355	HOM	≥37	≥37	≥37	≥37	≥37	≥37	<0.4	<0.4	<0.4	<0.4	<0.4	<0.4
370	HOM	≥37	≥37	≥37	≥37	≥37	≥37	<0.4	<0.4	<0.4	<0.4	<0.4	<0.4
380	HOM	≥37	≥37	≥37	≥37	≥37	≥37	<0.4	<0.4	<0.4	<0.4	<0.4	<0.4
388	HOM	≥37	≥37	≥37	≥37	≥37	≥37	<0.4	<0.4	<0.4	<0.4	<0.4	<0.4
408	HOM	≥37	≥37	≥37	≥37	≥37	≥37	<0.4	<0.4	<0.4	<0.4	<0.4	<0.4
438	HOM	≥37	≥37	≥37	≥37	≥37	≥37	<0.4	<0.4	<0.4	<0.4	<0.4	<0.4
442	HOM	≥37	≥37	≥37	≥37	≥37	≥37	<0.4	<0.4	<0.4	<0.4	<0.4	<0.4
487	HOM	≥37	≥37	≥37	≥37	≥37	≥37	<0.4	<0.4	<0.4	<0.4	<0.4	<0.4
267	HET	≥37	25.5	21.4	21.3	20.9	26.0	<0.4	<0.4	<0.4	<0.4	0.454	1.247
272	HET	≥37	24.6	19.5	22.0	24.2	27.7	<0.4	<0.4	<0.4	<0.4	0.485	0.925
282	HET	≥37	≥37	≥37	22.1	19.0	23.0	<0.4	<0.4	<0.4	<0.4	<0.4	0.954
357	HET	≥37	≥37	27.7	19.5	22.5	26.6	<0.4	<0.4	<0.4	<0.4	<0.4	<0.4
365	HET	≥37	≥37	≥37	≥37	≥37	19.0	<0.4	<0.4	<0.4	<0.4	<0.4	<0.4

389	HET	≥37	24.2	20.9	22.7	21.4	26.2	<0.4	<0.4	<0.4	0.434	0.474	0.680
400	HET	≥37	≥37	≥37	≥37	≥37	20.0	<0.4	<0.4	<0.4	<0.4	<0.4	<0.4
405	HET	≥37	28	19.1	19.8	21.0	26.2	<0.4	<0.4	<0.4	<0.4	0.485	0.554
288	NULL	≥37	≥37	≥37	30.3	22.2	Dead	<0.4	<0.4	<0.4	<0.4	<0.4	Dead
338	NULL	≥37	≥37	≥37	>=37	29.1	21.9	<0.4	<0.4	<0.4	<0.4	<0.4	0.411
359	NULL	≥37	≥37	≥37	≥37	≥37	20.7	<0.4	<0.4	<0.4	<0.4	<0.4	<0.4
377	NULL	≥37	≥37	≥37	27.5	22.1	25.1	<0.4	<0.4	<0.4	<0.4	<0.4	1.353
281	NULL	≥37	≥37	≥37	≥37	23.5	22.3	<0.4	<0.4	<0.4	<0.4	<0.4	<0.4

Table S4. Summary of PCR and ELISA results by zygosity in a room with pigs inoculated with PRRSV 1-8-4 L1H. Results are expressed as individual values before inoculation (day -1) and post-inoculation (days 3-21). PCR Ct values < 37 and ELISA S/P ratios ≥ 0.4 are positive. HOM = homozygous edited (*CD163*$^{\Delta E7 / \Delta E7}$), HET = heterozygous (*CD163*$^{\Delta E7 / +}$), and NULL = unedited null segregants (*CD163*$^{+ / +}$). Darker shaded areas show positive PCR and ELISA results, and lighter shaded areas show “suspect” results that can’t be confidently confirmed positive.

Pig ID	Zygosity	PCR (Ct < 37) Days Post-inoculation						ELISA (S/P ≥ 0.4) Days Post-inoculation					
		**-1**	**3**	**7**	**10**	**14**	**21**	**0**	**3**	**7**	**10**	**14**	**21**
841	HOM	≥ 37	≥ 37	≥ 37	≥ 37	≥ 37	≥ 37	<0.4	<0.4	<0.4	<0.4	<0.4	<0.4
835	HOM	≥ 37	≥ 37	35.4	≥ 37	≥ 37	≥ 37	<0.4	<0.4	<0.4	<0.4	<0.4	<0.4
866	HOM	≥ 37	≥ 37	≥ 37	≥ 37	≥ 37	≥ 37	<0.4	<0.4	<0.4	<0.4	<0.4	<0.4
883	HOM	≥ 37	≥ 37	≥ 37	≥ 37	≥ 37	≥ 37	<0.4	<0.4	<0.4	<0.4	<0.4	<0.4
887	HOM	≥ 37	≥ 37	≥ 37	≥ 37	≥ 37	≥ 37	<0.4	<0.4	<0.4	<0.4	<0.4	<0.4
815	NULL	≥ 37	18.4	15.3	17.5	192	22.2	<0.4	<0.4	<0.4	0.525	0.665	0.737
817	NULL	≥ 37	18.4	16.2	19.3	17.9	24.4	<0.4	<0.4	<0.4	1.177	1.123	1.183
711	NULL	≥ 37	22	18.3	17.5	17.7	24.1	<0.4	<0.4	<0.4	<0.4	0.55	0.55
865	NULL	≥ 37	19.3	14.8	18.7	20.9	24.2	<0.4	<0.4	<0.4	0.87	0.622	0.765
906	NULL	≥ 37	16.1	14	18.7	18.1	22.5	<0.4	<0.4	<0.4	0.853	0.661	1.138

Table S5. Summary of PCR and ELISA results by zygosity in a room with pigs inoculated with PRRSV 1-7-4 L1A. Results are expressed as individual values before inoculation (day -1) and post-inoculation (days 3-21). PCR Ct values < 37 and ELISA S/P ratios ≥ 0.4 are positive. HOM = homozygous edited (*CD163*$^{\Delta E7 / \Delta E7}$), HET = heterozygous (*CD163*$^{\Delta E7 / +}$), and NULL = unedited null segregants (*CD163*$^{+ / +}$). Darker shaded areas show positive PCR and ELISA results.

**Pig ID**	**Zygosity**	**PCR (Ct < 37) Days Post-inoculation**						**ELISA (S/P ≥ 0.4) Days Post-inoculation**					
		**-1**	**3**	**7**	**10**	**14**	**21**	**0**	**3**	**7**	**10**	**14**	**21**
836	HOM	≥ 37	≥ 37	≥ 37	≥ 37	≥ 37	≥ 37	<0.4	<0.4	<0.4	<0.4	<0.4	<0.4
770	HOM	≥ 37	≥ 37	≥ 37	≥ 37	≥ 37	≥ 37	<0.4	<0.4	<0.4	<0.4	<0.4	<0.4
862	HOM	≥ 37	≥ 37	≥ 37	≥ 37	≥ 37	≥ 37	<0.4	<0.4	<0.4	<0.4	<0.4	<0.4
890	HOM	≥ 37	≥ 37	≥ 37	≥ 37	≥ 37	≥ 37	<0.4	<0.4	<0.4	<0.4	<0.4	<0.4
892	HOM	≥ 37	≥ 37	≥ 37	≥ 37	≥ 37	≥ 37	<0.4	<0.4	<0.4	<0.4	<0.4	<0.4
828	NULL	≥ 37	15.8	12.3	21.5	16.8	29.2	<0.4	<0.4	<0.4	0.462	0.498	0.63
717	NULL	≥ 37	16	15.4	16.6	21	26.3	<0.4	<0.4	<0.4	0.505	<0.4	0.627
710	NULL	≥ 37	16	16.1	17.3	20.5	27.5	<0.4	<0.4	<0.4	<0.4	<0.4	<0.4
864	NULL	≥ 37	18.1	14.1	20.2	17.5	22.9	<0.4	<0.4	0.652	1.358	0.998	1.077
745	NULL	≥ 37	16.5	16.4	18	17.6	25.9	<0.4	<0.4	<0.4	0.517	<0.4	<0.4

Table S6. Summary of PCR and ELISA results by zygosity in a room with pigs inoculated with PRRSV 1-4-2 L1E. Results are expressed as individual values before inoculation (day -1) and post-inoculation (days 3-21). PCR Ct values < 37 and ELISA S/P ratios ≥ 0.4 are positive. HOM = homozygous edited (*CD163*$^{\Delta E7 / \Delta E7}$), HET = heterozygous (*CD163*$^{\Delta E7 / +}$), and NULL = unedited null segregants (*CD163*$^{+ / +}$). Darker shaded areas show positive PCR and ELISA results.

**Pig ID**	**Zygosity**	**PCR (Ct < 37) Days Post-inoculation**						**ELISA (S/P ≥ 0.4) Days Post-inoculation**					
		**-1**	**3**	**7**	**10**	**14**	**21**	**0**	**3**	**7**	**10**	**14**	**21**
842	HOM	≥ 37	≥ 37	≥ 37	≥ 37	≥ 37	≥ 37	<0.4	<0.4	<0.4	<0.4	<0.4	<0.4
773	HOM	≥ 37	≥ 37	≥ 37	≥ 37	≥ 37	≥ 37	<0.4	<0.4	<0.4	<0.4	<0.4	<0.4
858	HOM	≥ 37	≥ 37	≥ 37	≥ 37	≥ 37	≥ 37	<0.4	<0.4	<0.4	<0.4	<0.4	<0.4
891	HOM	≥ 37	≥ 37	≥ 37	≥ 37	≥ 37	≥ 37	<0.4	<0.4	<0.4	<0.4	<0.4	<0.4
893	HOM	≥ 37	≥ 37	≥ 37	≥ 37	≥ 37	≥ 37	<0.4	<0.4	<0.4	<0.4	<0.4	<0.4
824	NULL	≥ 37	26.5	20.3	21.5	23	27	<0.4	<0.4	<0.4	0.418	0.495	<0.4
716	NULL	≥ 37	28.1	20.8	20.9	20.5	26.4	<0.4	<0.4	<0.4	0.552	1.794	1.717
870	NULL	≥ 37	25.1	22.1	27.5	24.4	28.9	<0.4	<0.4	<0.4	1.328	1.432	1.539
861	NULL	≥ 37	27.8	21.2	26.7	26.5	29.1	<0.4	<0.4	<0.4	1.095	1.164	1.224
744	NULL	≥ 37	23.6	17.9	20.9	22	25.4	<0.4	<0.4	<0.4	0.605	1.025	1.005

Table S7. Depression and respiratory score of 1 by zygosity and expressed as total numbers and as a percentage. The number of observations by zygosity represents the sum of daily observations between challenge and 21 days post inoculation. Only scores of 0 and 1 were observed throughout the study after PRRSV inoculation, while scores of 2 and 3 were not observed. HOM = homozygous edited (*CD163*$^{\Delta E7 / \Delta E7}$), HET = heterozygous (*CD163*$^{\Delta E7 / +}$), and NULL = unedited null segregants (*CD163*$^{+ / +}$).

**Isolate**	**Zygosity (n=number)**	**Number of Observations with Depression Assessment Score of 1 (percentage)**	**Number of Observations with Respiratory Assessment Score of 1 (percentage)**
1-4-4 L1C	HOMO (n=286)<br>HET (n=176)<br>NULL (n=81)	3 (1.04 %)<br>24 (13.63 %)<br>27 (33.33 %)	7 (2.45 %)<br>23 (13.08 %)<br>14 (17.28 %)
NVSL97	HOMO (n=286)<br>HET (n=154)<br>NULL (n=110)	6 (2.09 %)<br>10 (6.49 %)<br>7 (6.36 %)	24 (8.39 %)<br>15 (9.74 %)<br>6 (5.45 %)
SD13-15	HOMO (n=264)<br>HET (n= 176)<br>NULL (n=103)	1 (0.37 %)<br>3 (1.70 %)<br>2 (1.94 %)	8 (2.99 %)<br>8 (4.59 %)<br>7 (6.79 %)

Figure S1 (A-C). Daily rectal temperatures from day 0 (pre-inoculation) to 21 days post-inoculation with PRRSV isolates: **(A)** 1-4-4 L1C, **(B)** NVSL97, and **(C)** SD13-15. The results are shown as average temperatures by zygosity. All temperatures were taken at the same time (am). A blue line separates temperatures higher or lower than 40oC. There were 13 homozygous pigs and 12 controls (eight heterozygous and four nulls) inoculated with 1-4-4 L1C; 13 homozygous and 12 controls (7 heterozygous and five nulls inoculated with NVSL97; and 13 homozygous pigs and 13 controls (8 heterozygous and five null pigs) inoculated with SD13-15. The tables below the figures show differences between zygosities, where different superscripts by day represent significant statistical differences (highlighted in shaded boxes).

A.

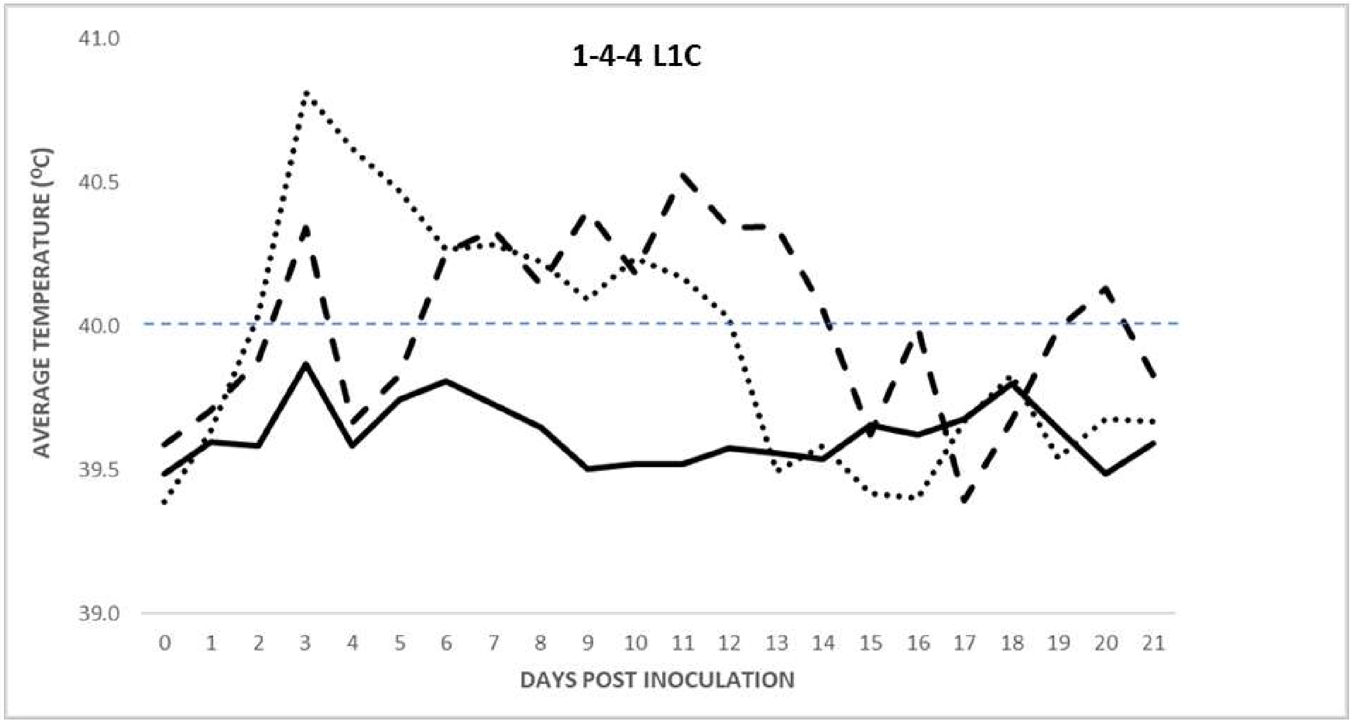

	0	1	2	3	4	5	6	7	8	9	10	11	12	13	14	15	16	17	18	19	20	21
HOM	39.5^{a}	39.6^{a}	39.6^{a}	39.9^{a}	39.6^{a}	39.7^{a}	39.8^{a}	39.7^{a}	39.6^{a}	39.5^{a}	39.5^{a}	39.5^{a}	39.6^{a}	39.6^{a}	39.5^{a}	39.7^{a}	39.6^{a}	39.7^{a}	39.8^{a}	39.6^{a}	39.5^{a}	39.6^{a}
NULL	39.6^{a}	39.7^{a}	39.9^{a}	40.3^{ab}	39.7^{a}	39.8^{bc}	40.3^{a}	40.3^{bc}	40.1^{ab}	40.4^{bc}	40.2^{bc}	40.5^{a}	40.3^{bc}	40.3^{b}	40.1^{a}	39.6^{a}	40.0^{a}	39.4^{a}	39.7^{a}	40.0^{a}	40.1^{b}	39.8^{a}
HET	39.4^{a}	39.6^{a}	40.0^{a}	40.8^{b}	40.6^{b}	40.5^{b}	40.3^{a}	40.3^{b}	40.2^{b}	40.1^{c}	40.2^{c}	40.2^{a}	40.0^{b}	39.5^{ac}	39.6^{a}	39.4^{a}	39.4^{a}	39.7^{a}	39.8^{a}	39.5^{a}	39.7^{ab}	39.7^{a}

B.

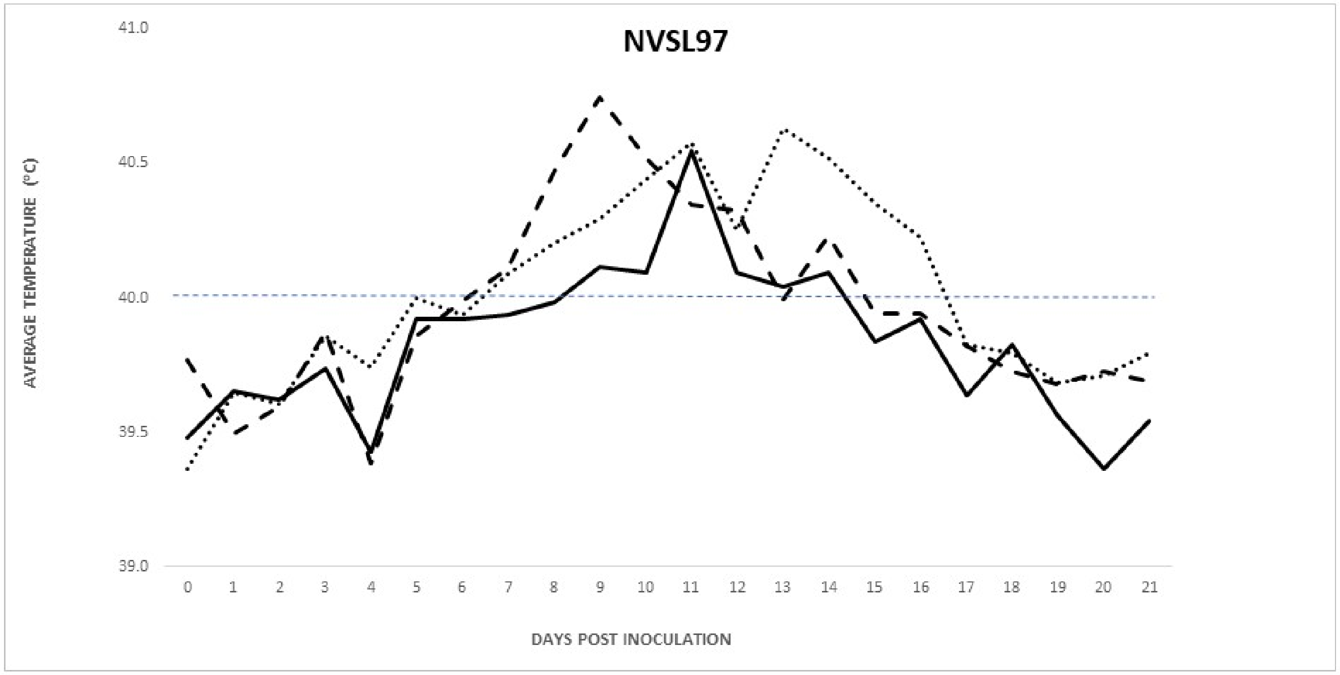

	0	1	2	3	4	5	6	7	8	9	10	11	12	13	14	15	16	17	18	19	20	21
HOM	39.3^{a}	39.6^{a}	39.6^{a}	39.7a	39.7^{a}	39.7^{a}	39.8^{a}	39.9^{a}	39.2^{a}	39.9^{a}	39.9^{a}	39.7 ^{a}	39.6^{a}	39.5^{a}	39.6^{a}	39.7^{a}	39.7^{a}	39.4^{a}	39.5^{a}	39.4^{a}	39.3^{a}	39.5^{a}
NULL	39.5^{a}	39.7^{a}	39.6^{a}	39.8^{a}	39.8^{a}	39.8^{a}	40.0^{a}	39.8^{a}	39.6^{a}	39.6^{a}	39.8^{b}	39.9^{a}	39.5^{a}	39.7^{a}	39.5^{a}	39.5^{ab}	39.3^{a}	39.9^{a}	39.5^{a}	39.6^{a}	39.5^{bc}	39.6^{a}
HET	39.5^{a}	39.8^{a}	39.5^{a}	39.6^{a}	39.7^{a}	39.6^{a}	39.9^{a}	39.7^{a}	39.5^{a}	39.6^{a}	39.9^{ab}	40.0^{a}	39.9a^{a}	39.7^{a}	39.6^{a}	39.8^{b}	39.6^{a}	39.7^{a}	39.4^{a}	39.7^{a}	39.6^{b}	39.7^{a}

C.

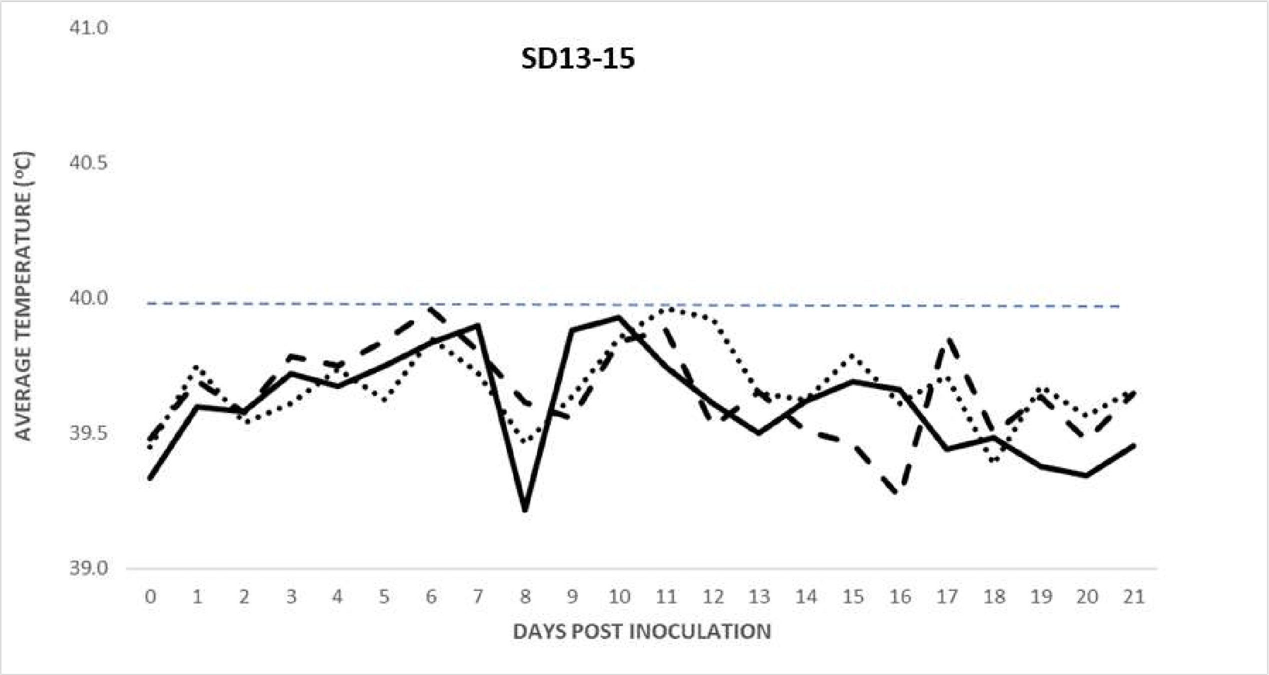

	0	1	2	3	4	5	6	7	8	9	10
HOM	39.3^{a}	39.6^{a}	39.6^{a}	39.7^{a}	39.7^{a}	39.7^{a}	39.8^{a}	39.9^{a}	39.2^{a}	39.9^{a}	39.9^{a}
NULL	39.5^{a}	39.7^{a}	39.6^{a}	39.8^{a}	39.8^{a}	39.8^{a}	40.0^{a}	39.8^{a}	39.6^{a}	39.6^{a}	39.8^{a}
HET	39.5^{a}	39.8^{a}	39.5^{a}	39.6^{a}	39.7^{a}	39.6^{a}	39.9^{a}	39.7^{a}	39.5^{a}	39.6^{a}	39.9^{a}

	11	12	13	14	15	16	17	18	19	20	21
HOM	39.7^{a}	39.6^{a}	39.5^{a}	39.6^{a}	39.7^{a}	39.7^{a}	39.4^{a}	39.5^{a}	39.4^{a}	39.3^{a}	39.5^{a}
NULL	39.9^{a}	39.5^{a}	39.7^{a}	39.5^{a}	39.5^{a}	39.3^{a}	39.9^{b}	39.5^{a}	39.6^{a}	39.5^{a}	39.6^{a}
HET	40.0^{a}	39.9^{a}	39.7^{a}	39.6^{a}	39.8^{a}	39.6^{a}	39.7^{ab}	39.4^{a}	39.7^{a}	39.6^{a}	39.7^{a}
